# *Aeromonas salmonicida* subsp. *salmonicida* in the light of its type-three secretion system

**DOI:** 10.1111/1751-7915.12091

**Published:** 2013-10-07

**Authors:** Philippe Vanden Bergh, Joachim Frey

**Affiliations:** Institute of Veterinary Bacteriology, University of BernLänggassstrasse 122, Bern, Switzerland

## Abstract

*Aeromonas salmonicida* subsp. *salmonicida* is an important pathogen in salmonid aquaculture and is responsible for the typical furunculosis. The type-three secretion system (T3SS) is a major virulence system. In this work, we review structure and function of this highly sophisticated nanosyringe in *A. salmonicida*. Based on the literature as well as personal experimental observations, we document the genetic (re)organization, expression regulation, anatomy, putative functional origin and roles in the infectious process of this T3SS. We propose a model of pathogenesis where *A. salmonicida* induces a temporary immunosuppression state in fish in order to acquire free access to host tissues. Finally, we highlight putative important therapeutic and vaccine strategies to prevent furunculosis of salmonid fish.

## Introduction

The disease furunculosis caused by *Aeromonas salmonicida* subsp. *salmonicida* (hereafter referred to as *A. salmonicida*) continues to be a major health problem for the growing salmonid aquaculture worldwide. The disease, known for over a century, results in significant economic losses and promotes the intensive use of antibiotics. In spite of effective commercialized oil-adjuvanted vaccines containing *A. salmonicida* bacterins, frequent outbreaks persist at fish farms. Different virulence factors of *A. salmonicida* have been described but they failed to explain the virulence phenotype of the pathogen (Ellis *et al*., 1988; Vipond *et al*., 1998). Ten years ago, our laboratory published the first descriptions of the type-three secretion system (T3SS) in the genus *Aeromonas* and demonstrated its role as a main virulence secretion system of *A. salmonicida* (Burr *et al*., 2002; 2003b; Stuber *et al*., 2003). Here, we provide the first review on this highly sophisticated nanosyringe and its putative role in a model of pathogenesis for typical furunculosis.

## Genetic organization of the *A. salmonicida* T3SS

The structural components of *A. salmonicida* T3SS are encoded on a large conjugative plasmid of 140–155 kb (Stuber *et al*., 2003; Reith *et al*., 2008) by numerous genes arranged in five predicted polycistronic operons (*exsADascBCDEFGHIJKL*, *exsCEB*, *aopNacr12ascXYVacrRGVHaopBD*, *ascNOPQRSTU* and *aopXsycX*) (Fig. S1). The plasmid pASA5 of 155 kb has been entirely sequenced in the reference strain *A. salmonicida* A449. The structural genes are flanked on both sides by genes encoding for T3SS effectors and their chaperones (*ati1ati2*, *aopHsycH*, *aopOsycO* and putatively *asa_P5G088*) interspaced by various kinds of insertion elements (IS*256*, IS*630*, IS*116*) (Fig. S1) (Reith *et al*., 2008). The role of these insertion elements (ISs) in the genetic rearrangements of the T3SS loci is crucial. For example, T3SS cluster and other part of the pASA5 plasmid are lost when *A. salmonicida* is cultivated in stressful conditions, and it has been demonstrated that this deletion process is due to the homologous recombination between IS*256* copies which are present at the extremities of the T3SS cluster (Daher *et al*., 2011; Tanaka *et al*., 2012; 2013) (Fig. S1). More complex rearrangements involving other ISs are also suspected. IS*630* copies might also play a role in the genetic organization of the injectisome because they are frequently associated with T3SS loci in *A. salmonicida* (Studer *et al*., 2013) (Fig. S1) but also in other pathogenic bacteria using such secretion systems (Haneda *et al*., 2001; Correa *et al*., 2012; Stavrinides *et al*., 2012). The cluster of T3SS genes can also be integrated in the chromosome of some strains of certain species of *Aeromonas*. Such an insertion is observed in *A. hydrophila* strain SSU, *A. veronii* strain AER39 and *A. diversa* 2478–85 (Fig. S1, Table S1). The *A. diversa* 2478–85 strain is currently the only *Aeromonas* strain showing a second T3SS-2 similar to the one of *Edwardsiella tarda* (Wang *et al*., 2010), directly adjacent to the T3SS shared by several *Aeromonas* species (Fig. S1, Table S1) and associated to other T3SS virulence effectors such as ExoU described in *Pseudomonas aeruginosa* (Shaver and Hauser, 2004) (Fig. S1, Table S1). This observation demonstrates that T3SS gene clusters from other bacterial genera can be integrated in the *Aeromonas* genome.

The *aopH* gene has also been shown to be encoded together with its chaperone on pASA6, a smaller plasmid of 18.5 kb, which has a high identity with parts of pASA5 suggesting that pASA6 is a derivative of this latter plasmid. The effector gene *aopP* has been detected on pAsal1, a small plasmid of 6.4 kb (Fig. S1) which shows some identity with pASA3 (*aopP* negative), another small plasmids. The pAsal1 plasmid seems also to be sensitive to stressful growth conditions (Tanaka *et al*., 2012). Another large conjugative plasmid of 167 kb is pASA4 which is homologous to plasmid pRA1 and pRA3 of *A. hydrophila* and contains genes associated with resistance to antibiotics and numerous undetermined coding sequences but no known coding sequence for T3SS elements. The genes of the adenosin diphosphate (ADP) ribosylating toxin AexT and AopS (ASA_0010, homologous to VopS of *Vibrio parahaemolyticus*), both T3SS effectors, and their chaperones are to date the only ones detected on the chromosome of *A. salmonicida* A449 (Fig. S1). It has, however, to be noted that *aopS* is predicted to be a pseudogene because of an in-frame stop codon (Reith *et al*., 2008). An intact copy of *aopS* and the gene for its chaperone are present at the same position in the chromosome of *A. salmonicida* subsp. *achromogenes* strain AS03 (Table S1). The prevalence of intact *aopS* in other *A. salmonicida* strains is not known. Interestingly, among the different published genomes of *Aeromonas* sp. published, *aexT* and its chaperone (*sycE*) are always localized on the same genomic island present between *glyS* (*asa_4261*) and the transcriptional regulator *asa_4278* (Table S1) which shows variability between strains from different *Aeromonas* species. *Aeromonas salmonicida* A449 and 01-B526, *A. hydrophila* SSU and *A. veronii* AER39 detain the *aexTsycE* cluster at this position whereas *A. hydrophila* ATCC7966; *A. caviae* Ae398; *A. veronii* B565, AMC34, AMC35 and AER397; and *A. aquariorum* AAK1 do not contain these loci (Table S1). The presence of *aexTsycE* at the same position in the genome of different *Aeromonas* species likely suggests that they would be inherited from an ancestor and lost in some strains or that they are integrated by a process specifically targeting this site.

Therefore, while the entire genetic pattern of *A. salmonicida* subsp. *salmonicida* strains is well conserved worldwide and over time (Studer *et al*., 2013), the cloud of virulence genes associated with the T3SS is constantly submitted to local genetic deletions in specific constraints and genetic additions through the exchanges by horizontal transfer of genes with other environmental bacteria (Burr and Frey, 2007). These processes of gene deletions might be one of the reasons for the progressive loss of virulence observed with *A. salmonicida* laboratory strains that are intensively cultivated under conditions that, without the selection pressure they are subjected to in the host, are unsuitable to preserve virulence. A typical example is the type strain of *A. salmonicida* ATCC 33658T that has lost all T3SS structural genes and is non-virulent (Burr and Frey, 2009). These genetic rearrangements highlight why it is a real challenge to work with *A. salmonicida* to obtain relevant data on the pathogenesis. Unless strict conditions of culture are respected, it is possible for genetic modifications to occur in the time-lapse between the isolation of the bacterium and its use for genetic characterization (molecular epidemiology) and molecular manipulations (site-directed mutagenesis). Furthermore, molecular epidemiologic studies are of concern as they take into account the presence or absence of only few T3SS genes to draw conclusions on the virulence of *A. salmonicida* strains. Because of the numerous mutations and genetic rearrangements affecting the *A. salmonicida* T3SS, epidemiological studies should include the analysis of the integrity of several genes for T3SS structural components (at least *ascV* and *ascC*) and the genes of the main effectors (*aopH*, *aexT*, *ati2*, *aopO*, *aopP* and *aopS*) (Burr and Frey, 2007; Daher *et al*., 2011) in order to draw conclusions regarding the presence of an intact T3SS with functional effectors.

## Regulation of the *A. salmonicida* T3SS expression

**Figure 1:**
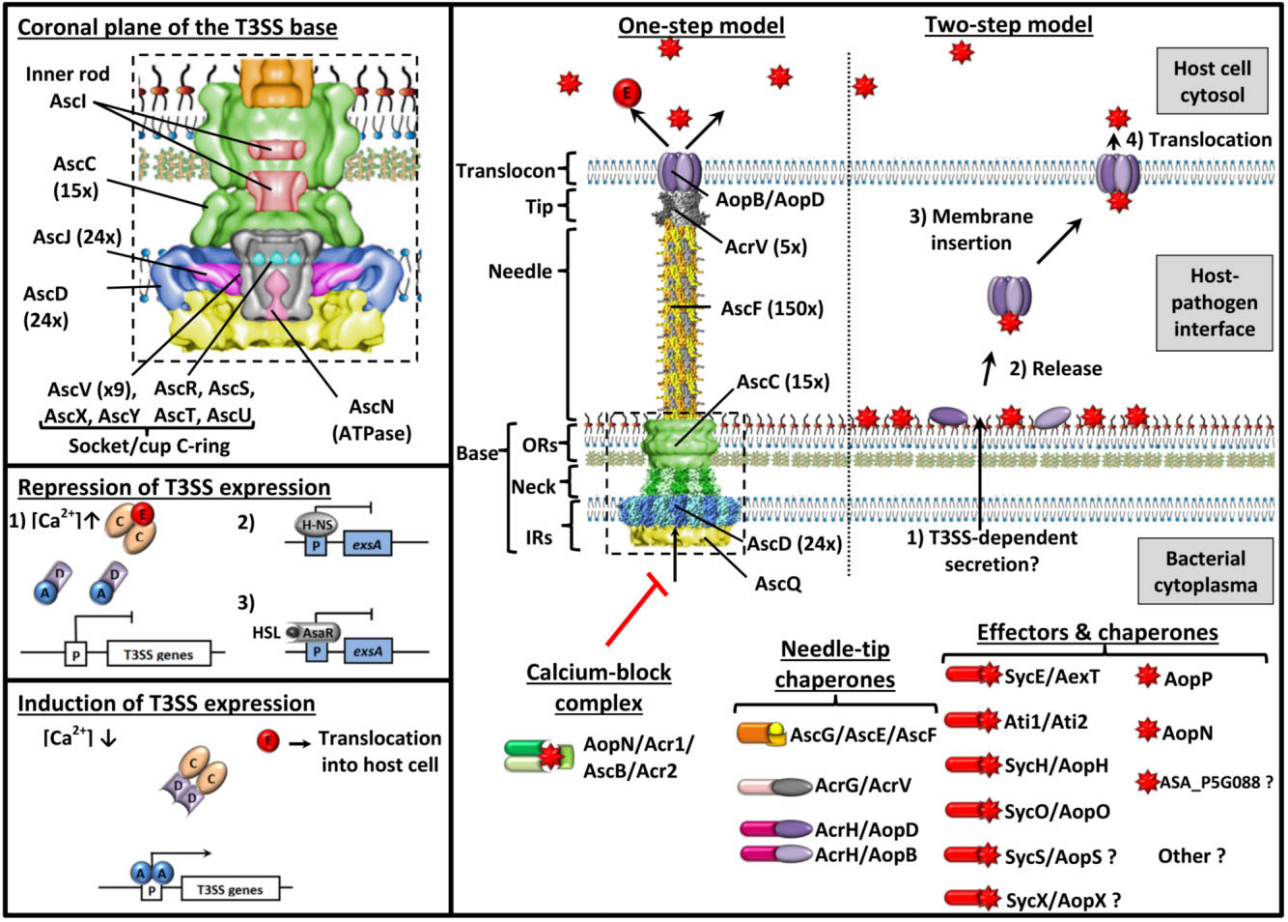
*A. salmonicida* T3SS transcription regulation, anatomy and models of effector translocation into host cell. Negative (ExsABDCE, Hns and quorum sensing) and positive (ExsABCDE) pathways regulating the transcriptional expression of the *A. salmonicida* T3SS are depicted in the two boxes at the bottom left. The structural components, chaperones, effectors and secretion regulators of the *A. salmonicida* T3SS and the two described models of effector translocation into fish cell are represented in the main box. The upper left box shows the structural components present inside the T3SS base. Under non-permissive conditions (for example with high extracellular calcium concentrations), the transcription of T3SS genes induces by the positive activator ExsA is expected to be inhibited by the formation of inhibitory ExsD-ExsA (1:1) and ExsC-ExsE (2:1) complexes (Brutinel and Yahr, 2008; Brutinel *et al*., 2009). Permissive conditions would activate the T3SS secretory activity and induce the secretion and translocation of ExsE. Histone-like proteins (H-NS) could repress T3SS gene expression by suppressing *exsA* gene expression like for *V. parahaemolyticus* (Kodama *et al*., 2010). In growing bacterial cultures, AsaR from the quorum sensing is activated by the binding of homoserinelactone (HSL) and could directly repress the expression of *exsA* and subsequently the expression of the T3SS like for *Vibrio* (Waters *et al*., 2010). The T3SS is a highly sophisticated nanosyringe which is mainly composed of Asc (*Aeromonas* secretion) proteins, the building blocks of the injectisome, and Aops (*Aeromonas* outer proteins) which are released outside of the bacterium (Hodgkinson *et al*., 2009; Diepold *et al*., 2011; 2012; Abrusci *et al*., 2013; Dewoody *et al*., 2013). In other bacterial species, the number of subunits in certain parts of the T3SS is known and is indicated in brackets. In the bacterial cytoplasma are represented the calcium-block complex which regulates the T3SS secretion and chaperones of the needle, the tip and effectors. Currently, two models of translocation into host cell (one-step vs two-step) are under debates (Perrett and Zhou, 2011).

The transcription of the T3SS genes are induced under Ca^2+^-limiting conditions (Burr *et al*., 2003a) and during the contact of *A. salmonicida* with the host cell (Braun *et al*., 2002). The transcription of the *A. salmonicida* T3SS genes is predicted to be controlled by a regulatory pathway similar to that observed for the T3SS of *P. aeruginosa* (Brutinel and Yahr, 2008; Brutinel *et al*., 2009). The mechanism involves four interacting regulatory proteins (ExsA, ExsD, ExsC and ExsE) (Fig. 1). ExsA is a positive activator of T3SS transcription, whereas ExsD is an anti-activator that forms a 1:1 complex with ExsA. Under non-permissive conditions for T3SS gene expression, ExsA-dependent transcription is expected to be inhibited by the formation of inhibitory ExsD-ExsA (1:1) and ExsC-ExsE (2:1) complexes. Permissive conditions would activate the T3SS secretory activity and induce the secretion and translocation of ExsE. The decrease of intracellular concentration of ExsE seems to promote the formation of ExsD-ExsC (2:2) complexes inducing the release of ExsA and the activation of T3SS gene expression. In *V. parahaemolyticus*, a histone-like protein (H-NS) represses T3SS gene expression by suppressing *exsA* gene expression (Kodama *et al*., 2010). This second level of transcriptional control is assumed in *A. salmonicida* given that several H-NS homologues are present in the chromosome and the pASA5 plasmid (ASA_P5G002 and ASA_P5G018). Another level of *A. salmonicida* T3SS expression control is performed by the quorum sensing (QS) pathways encoded at least by the genes *asaIR* and *luxS*, *luxU*, *luxO* (Reith *et al*., 2008) which produce high homoserinelactone (HSL) concentration in the extracellular medium at high cell density (Swift *et al*., 1997). Like for *Vibrio*, in growing bacterial cultures, AsaR is activated by the binding of HSL and could directly repress the expression of *exsA* and subsequently the expression of the T3SS (Waters *et al*., 2010). However, expression of *A. salmonicida* proteases and lipases is QS-induced at high cell density and is important to induce the virulence (Rasch *et al*., 2007; Schwenteit *et al*., 2011). These assumptions are supported by our proteomic analysis of the wild-type (wt) JF5054 strain of *A. salmonicida* (virulent reference strain) showing that the amount of T3SS decreases significantly from the exponential to the stationary phase of growth whereas the expression of some enzymes (proteases, lipases, chitinases, etc) increases. These observations also highlight that the T3SS activation state in the bacterial culture is a critical point to take into account for the preparation of the inoculum for experimental challenges with *A. salmonicida* besides the genetic variations and losses of virulence attributes. Bacterial preparations from late phases of growth with a downregulated T3SS appear to be associated with low mortality despite the genotypic presence of all the T3SS genes.

## Morphology of the *A. salmonicida* T3SS

**Figure 2:**
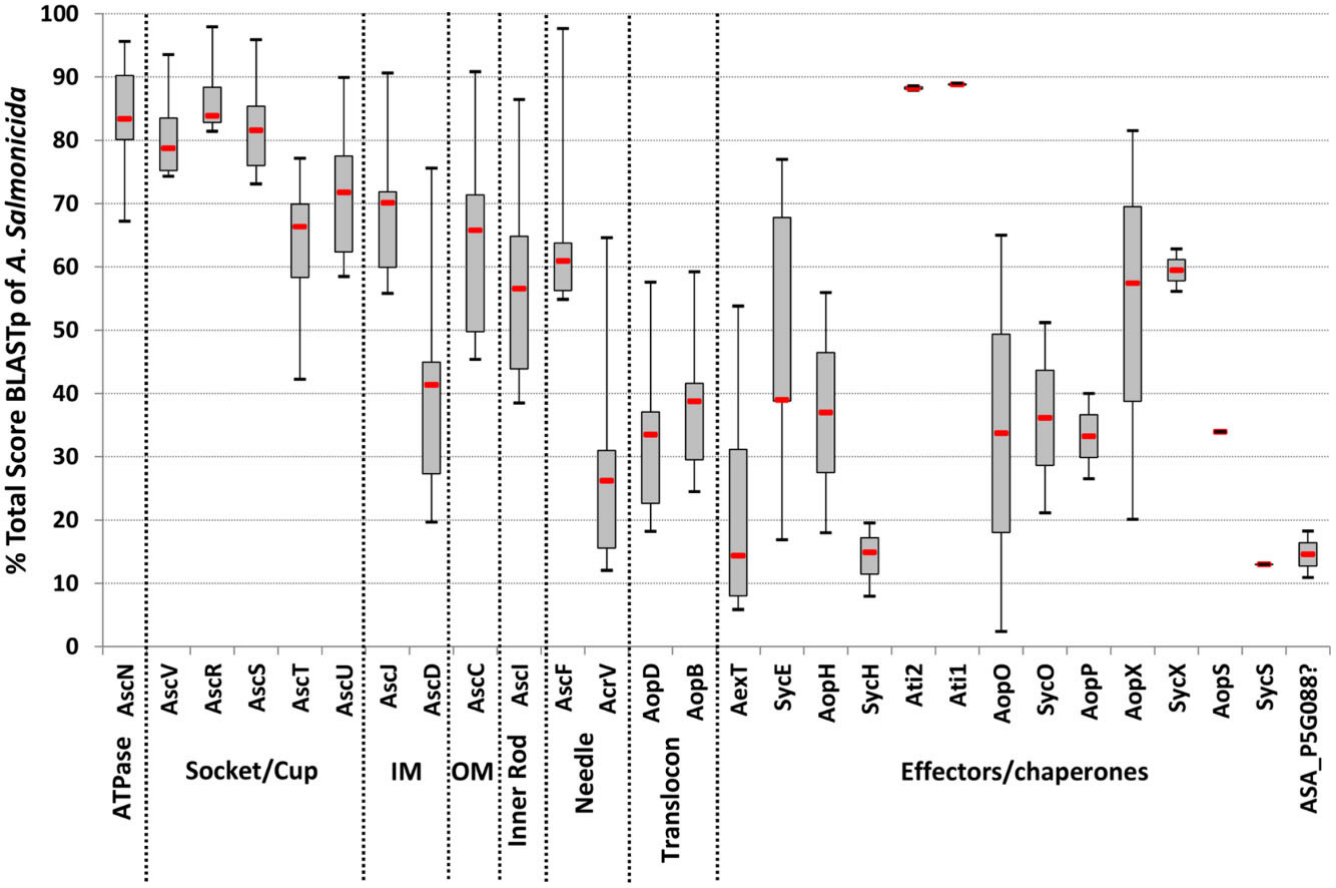
T3SS homology in the Ysc family. The diagram shows the conservation variability of T3SS structural components of *Photorhabdus luminescens*, Yersinia pestis, *Vibrio parahaemolyticus*, *Pseudomonas aeruginosa* and *Photobacterium damselae* in comparison to A. salmonicida subsp. salmonicida. For each cited T3SS component, the variability (BLASTp total score) of orthologues is represented in percentage of the A. salmonicida BLASTp total score. The homology decreases from the inner to the outer parts of the T3SS and is the lower for the translocon.

Currently, no structural studies have specifically been carried out on the T3SS of *A. salmonicida* but this secretion apparatus shows conservation with the well-described T3SS of *Yersinia* and *Salmonella* (Table 1). Hence, the following information about the morphology and the functioning of the *A. salmonicida* T3SS is inferred from structural homologues reviewed in reference articles (Hodgkinson *et al*., 2009; Diepold *et al*., 2011; 2012; Abrusci *et al*., 2013; Dewoody *et al*., 2013). Proteins orthologue to all of the *A. salmonicida* T3SS components are detailed in Table 1. The T3SS structural proteins of *Photorhabdus luminescens*, *Yersinia pestis*, *V. parahaemolyticus*, *P. aeruginosa* and *Photobacterium damselae* present the highest conservation with the *Aeromonas* T3SS and constitute the ‘Ysc’ family of T3SS (Barret *et al*., 2013) (Fig. S1). The T3SS is a highly sophisticated nanosyringe that is mainly composed of Asc (*Aeromonas* secretion) proteins, the building blocks of the injectisome, and Aops (*Aeromonas* outer proteins) which are released outside of the bacterium (Fig. 1). Interestingly, the degree of conservation of T3SS subunits between these bacteria is the highest for the membrane components and the lowest for the translocation complex suggesting that a more important evolution of the external parts of the T3SS may be related to host adaptation (Fig. 2). Some of the T3SS effectors are present in multiple genera with a low similarity while others (for example Ati2) are shared by some genera with a high similarity suggesting a recent horizontal gene transfer (Fig. 2 and Fig. S1).

**Table 1:** Proteins orthologue to *A. salmonicida* T3SS components

T3SS part	Information	Ysc Family				Ssa/Esc Family			
		*Aeromonas*	*Photorhabdus*	*Yersinia*	*Vibrio*	*Pseudomonas*	*Bordetella*	*Salmonella*	*Shigella*
Translocon	Pore	AopB	LopB	YopB	VopB	PopB	BopB	SipB	IpaB
	Pore	AopD	LopD	YopD	VopD	PopD	BopD	SipC	IpaC
	Chaperone	AcrH	LssH	SycD/LcrH	VcrH	PcrH	BcrH1	SicA	LpgC
Needle	Tip	AcrV	LssV	LcrV	VcrV	PcrV	Bsp22	SipD	IpaD
	Chaperone	AcrG	LssG	LcrG	VcrG	PcrG	–	–	–
	Body	AscF	LscF	YscF	VscF	PscF	BscF	PrgI	MxiH
	Chaperone	AscG	LscG	YscG	VscG	PscG	–	–	–
	Chaperone	AscE	LscE	YscE	VscE	PscE	BscE	–	–
	Chaperone	AcrR	LssR	LcrR	VcrR	PcrR	–	–	–
	Polymerization control	AscH	LscH	YscH/YopR	VscH	PscH	–	–	–
	Length control	AscP	LscP	YscP	VscP	PscP	BscP	InvJ	Spa32
Base	OM ring	AscC	LscC	YscC	VscC	PscC	BscC	InvG	MxiD
	Chaperone	AscW/ExsB	LscW	YscW	VscW	ExsB	–	–	–
	Inner rod	AscI	LscI	YscI	VscI	PscI	BscI	PrgJ	MxiI
	IM ring ext	AscD	LscD	YscD	VscD	PscD	BscD	PrgH	MxiG
	IM ring int	AscJ	LscJ	YscJ	VscJ	PscJ	BscJ	PrgK	MxiJ
	C-ring, socket/Cup	AscR	LscR	YscR	VscR	PscR	BscR	SpaP	Spa24
	C-ring, socket/Cup	AscS	LscS	YscS	VscS	PscS	BscS	SpaQ	Spa9
	C-ring, socket/Cup	AscT	LscT	YscT	VscT	PscT	–	SpaR	Spa29
	C-ring, socket/Cup	AscU	LscU	YscU	VscU	PscU	BscU	SpaS	Spa40
	C-ring, socket/Cup	AscV	LssD	YscV	VcrD	PcrD	BcrD	InvA	MxiA
	C-ring, secretion specificity	AscX	LssB	YscX	VscX	PscX/Pcr3	–	–	–
	C-ring, secretion specificity	AscY	LssC	YscY	VscY	PscY/Pcr4	Bcr4	–	–
	ATPase	AscN	LscN	YscN	VscN	PscN	BscN	InvC	Spa47
	Cytoplasmic	AscK	LscK	YscK	VscK	PscK	BscK	OrgA	MxiK
	Cytoplasmic	AscL	LscL	YscL	VscL	PscL	BscL	OrgB	MxiN
	Cytoplasmic	AscQ	LscQ	YscQ	VscQ	PscQ	BscQ	SpaO	Spa33
Regulators	Secretion	AopN	LopN	YopN	VopN	PopN	BopN	InvE	MxiC
	Secretion	Acr1	LssA	TyeA	Vcr1	Pcr1	–	–	–
	Secretion	AscB	LscB	YscB	VscB	PscB	–	–	–
	Secretion	Acr2	LssN	SycN	Vcr2	Pcr2	–	–	–
	Substrate recycling?	AscO	LscO	YscO	VscO	PscO	BscO	InvI	Spa13
	T3SS transcription	ExsA/AscA	LscA	LcrF	ExsA	ExsA	–	–	–
	T3SS transcription	ExsC	LscY	–	ExsC	ExsC	–	–	–
	T3SS transcription	ExsD	LscZ	–	ExsD	ExsD	–	–	–
	T3SS transcription	ExsE	ExsE	–	ExsE	ExsE	–	–	–
Effectors	ADP-ribosylase + GAP	AexT	–	YopE	VopT	ExoT+ExoS	–	–	–
	Chaperone	SycE	Plu3789	SycE/YerA	–	CesT+CesT	–	–	–
	Phosphotyrosine phosphatase	AopH	–	YopH	–	–	–	–	–
	Chaperone	SycH	–	SycH	–	–	–	–	–
	Inositol Phosphatase	Ati2	plu4615	–	VPA0450	–	–	–	–
	Chaperone	Ati1	Plu4614	–	VPA0451	–	–	–	–
	Serine/threonine kinase	AopO	–	YopO/YpkA	–	–	–	–	–
	Chaperone	SycO	–	SycO	–	–	–	–	–
	NF-*κ*B inhibition + apoptosis	AopP	–	YopJ/YopP	VopA/P	–	–	AvrA	–
	Lipid rafts?	AopX	Plu4750	–	VopR	–	BteA	–	–
	Chaperone	SycX	–	–	–	–	–	–	–
	AMPylation	AopS	–	–	VopS/VepB	–	–	–	–
	Chaperone	SycS	–	–	VPA0451	–	–	–	–
	Hydrolase	ASA_P5G088 ?	–	–	VP1677, −1678	–	–	–	–

The base of the T3SS (Fig. 1) consists of a series of rings which spans the bacterial membranes and the periplasmic space (AscD and AscJ form inner membrane rings and AscC constitutes the outer membrane ring). At the centre of the inner membrane rings, a socket/cup domain (AscR, AscS, AscT, AscU and AscV) is present and is the technical platform for the secretion of T3SS components. Numerous cytoplasmic components (AscK, AscL and AscQ) are recruited to this site to orchestrate an active and orderly secretion of various T3SS substrates by an ATPase (AscN). Inside the outer membrane ring, an inner rod (AscI) connects the socket/cup to the distal part of the needle. The needle (AscF) and its tip (AcrV) make the bridge between the bacterium and the host cell, and connect the translocon (AopB/AopD) which is inserted into the host cell membrane and activates the translocation of T3SS effectors into the eukaryotic cytosol according to the ‘one-step’ model of secretion (Perrett and Zhou, 2011) (Fig. 1).

Using hybrid T3SS in *Y. enterocolitica* by exchanging the gene *lcrV* encoding the *Yersinia* T3SS tip structure with *acrV*, the analogous gene of *A. salmonicida*, Broz and colleagues (2007) proved the functionality of AcrV as T3SS tip structure representing the base protein of the tip. In that experiment, tip complexes formed by AcrV were larger and were diverse in shape. Using hybrid LcrV/AcrV tip structures, the authors concluded that the N-terminal domain of LcrV and AcrV form the base of the tip complex while the central globular domains of the proteins form the head of the tips.

Beside these structural elements and the effectors, the T3SS possesses numerous regulatory proteins which play different roles (Fig. 1). Chaperones stabilize T3SS proteins in the bacterial cytosol and deliver them to the ATPase AscN for the active secretion (AcrH for AopB/AopD, AcrG for AcrV, AscG/AscE/AcrR for AscF, AscW for AscC and SycE/SycH/Ati1/SycO/SycS for associated effectors). Other regulatory proteins control the polymerization (AscH) and the length (AscP) of the needle, the opening of the conduit upon host cell contact (the calcium-block complex AopN/Acr1/AscB/Acr2) and the specificity of the secretion (AscX and AscY).

## The T3SS is the main virulence system of *A. salmonicida*

To date, several virulence factors have been characterized for *A*. *salmonicida* but the T3SS is currently the only one recognized as having a major effect on virulence. This was shown by independent studies with isogenic mutant strains for T3SS structural proteins which proved to be non-virulent both *in vitro* and *in vivo* (Burr *et al*., 2002; 2003b; 2005; Dacanay *et al*., 2006; Froquet *et al*., 2007; Daher *et al*., 2011). These results are exemplified by current results showing that the intraperitoneal (i.p.) injection of 500 cfu per fish of the fully virulent wt JF5054 strain (the JF2267 strain which was freshly reisolated from an experimentally infected dead fish) induces 70–80% of mortality whereas the isogenic T3SS-deficient mutant derivative (JF2747) deleted for *ascV* (protein of the socket/cup in the inner membrane) is considered to have extremely low virulence because i.p. injection of 10^5^ cfu/fish induced no mortality (Burr *et al*., 2005). Furthermore, 10^8^cfu/fish (200 000 times more than the inoculation with the wt strain), a drastic dose that does not reflect a natural infection, merely induced a weak mortality of only 20% (this work).

This phenotypic difference between the wt and the *ΔascV* mutant strains led us to compare the exoproteome of these bacteria by high-throughput proteomics (P. Vanden Bergh, unpublished) and established the full repertoire of *in vitro* T3SS effectors that are excreted in the supernatant (SN) by the wt strain of *A. salmonicida*. The other virulence factors described that are not part of the T3SS secretome (VapA, AerA, AerB, GCAT, Pla1, PlaC, TagA, Ahe2, GbpA and enolase) are expressed to a higher extent in the avirulent *ΔascV* mutant than in the wt strain and hence seem to play a secondary role in the pathogenesis.

**Figure 3:**
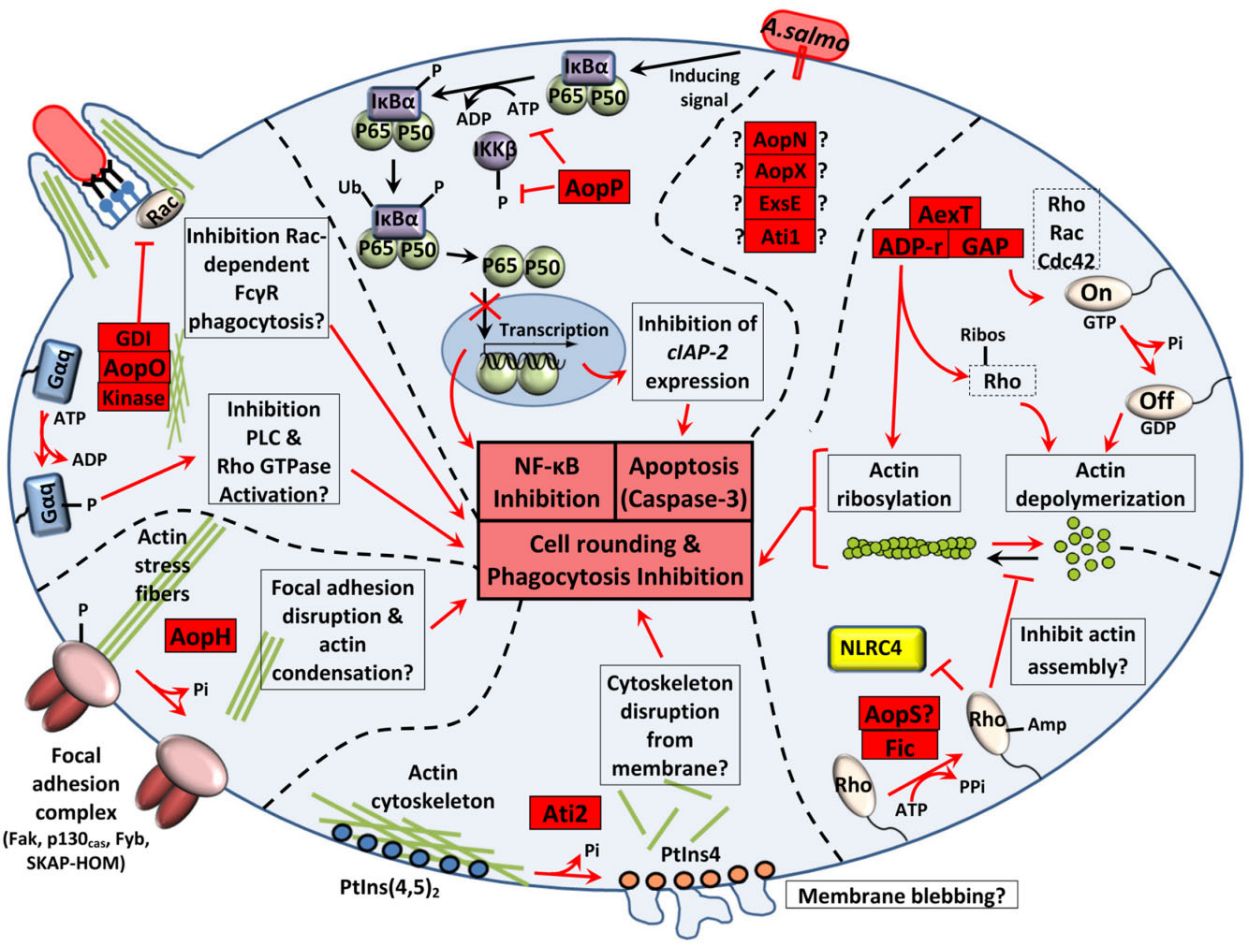
Main intracellular effects of *A. salmonicida* T3SS effectors. The intracellular effect of AexT, Ati2 and AopP has been characterized by previous studies while the impact of AopH, AopO and AopS is predicted from cytotoxic homologues from other bacterial species. AopN, AopX, ExsE, and Ati1 might also be translocated into the host cell but their role in virulence is not known. AopS and AopX are encoded by pseudogenes in the reference A449 strain. AexT, AopS, Ati2, AopH, and AopO would exert different negative effects on cytoskeletal dynamics. T3SS effectors are represented by red boxes with their known or predicted functional domains: ADP-ribosylating (ADP-r) and GTPase-activating (GAP) domains of AexT, filamentation induced by cAMP (Fic) domain of AopS, and the guanine-nucleotide dissociation inhibitor (GDI)-like domain of AopO. cIAP-2: cellular inhibitor of apoptosis 2; I*κ*B*α*: inhibitor of the nuclear transcription factor NF-*κ*B; IKK*β*: I*κ*B kinase *β*; Fc*γ*R: Fc-gamma receptor; Gαq: the heterotrimeric G protein subunit Gα_q_; NLRC4: Nod Like Receptor family CARD domain-containing protein 4, PLC: phospholipase C; PtIns: phosphatidylinositol; Rho, Rac, and Cdc42: GTPases of the Rho family.

The T3SS arsenal of *A. salmonicida* is mainly composed by AexT, AopH, Ati2, AopP, AopO, AopN and ExsE. AopS and AopX were not identified in our analysis but are potential T3SS effectors that could be expressed by *A. salmonicida* strains possessing functional copy of their genes. The occurrence of deletions in the gene of *aopS* and *aopX* effector genes suggests that *A. salmonicida* undergoes a pressure of selection to lose their function. Only the effect of AexT, AopP and Ati2 has been characterized in the cytotoxicity induced by *A. salmonicida* (Fig. 3). However, the other effectors show similarity with T3SS effectors studied in other pathogenic bacteria (Dean, 2011) and their function can thus be predicted (Fig. 3).

The bifunctional toxin **AexT** possesses a GTPase-activating domain acting on small monomeric GTPases of the Rho family (Rho, Rac and Cdc42) and an ADP-ribosylating domain, which ADP-ribosylates both muscular and non-muscular actin (Braun *et al*., 2002; Burr *et al*., 2003a; Fehr *et al*., 2007). Both enzymatic domains play an independent role in the actin depolymerization and cell rounding (Fehr *et al*., 2007).

**AopH** is similar to *Yersinia* phosphotyrosine phosphatase YopH which dephosphorylates tyrosine residues of protein in focal adhesion complexes at the cellular membrane resulting in loss of focal adhesion complex, alteration of the actin cytoskeleton and blocking of phagocytosis (Cornelis, 2002; Broberg and Orth, 2010). In *Yersinia*, this effector inhibits also the lymphocyte proliferation and the synthesis of monocyte chemotactic protein 1 (Cornelis, 2002).

**Ati2** is a phosphatidylinositol (PtIns) phosphatase that hydrolyses the D5 phosphate from PtIns(4,5)P_2_ and PtIns(3,4,5)P_3_ (Dallaire-Dufresne *et al*., 2013). This effector is homologous to VPA0450 of *Vibrio parahaemolyticus* which induces the local detachment of the actin-binding proteins from the plasma membrane, causes membrane blebbing and accelerates cytolysis by removing PtIns(4,5)P_2_ (Broberg *et al*., 2010).

**AopP** inhibits the NF-*κ*B signalling pathway by preventing the translocation of the p50/p65 protein complex (NFKB1/RelA) into the nucleus of target cells (Fehr *et al*., 2006). AopP inhibits I*κ*B kinase *β* (IKK*β*) phosphorylation and attenuates I*κ*B*α* phosphorylation showing that AopP-mediated inhibition of NF-*κ*B occurs upstream of I-*κ*B phosphorylation. This NF-*κ*B pathway inhibition is highly proapoptotic upon concurrent tumor necrosis factor-α cellular stimulation (Jones *et al*., 2012). Apoptosis would be induced in part by the subsequent inhibition of the expression of anti-apoptotic factors (IAP) (Jones *et al*., 2012). AopP activity is unable to inhibit mitogen-activated protein kinase pathways (such as ERK, p38 and JNK) contrary to its orthologues YopJ, VopA and AvrA (Jones *et al*., 2012).

**AopO** is related to the *Yersinia* YopO/YpkA, a serine/threonine kinase that disrupts the normal distribution of actin in the host cell (Nejedlik *et al*., 2004). The kinase domain localizes the effector to the plasma membrane and is activated by actin binding. It phosphorylates and inactivates Gα_q_ signalling pathways affecting multiple downstream targets (Broberg and Orth, 2010). Moreover, this effector inhibits phagocytosis through its guanine-nucleotide dissociation inhibitor-like domain by specifically blocking Rac-dependent Fc*γ* receptor internalization pathway (Groves *et al*., 2010).

**AopN** homologues in other bacteria (HrpJ and BopN) are T3SS effectors which play a role in virulence and can have a dual role: controlling the secretion of translocator proteins inside bacteria and suppressing immunity when AopN is translocated inside host cells (Nagamatsu *et al*., 2009; Crabill *et al*., 2012). In *Chlamydia*, translocated CopN, the analogue to AopN, is able to bind and sequester αβ-tubulin and inhibits microtubule polymerization leading to the loss of microtubule spindles and metaphase plate formation inducing mitotic arrest (Archuleta *et al*., 2011).

**AopS** is homologous to *V. parahaemolyticus* VopS which induces the AMPylation of Rho GTPases through its Fic domain (Yarbrough *et al*., 2009). This inhibition prevents the interaction of Rho GTPases with downstream effectors, thereby inhibiting actin assembly (Yarbrough *et al*., 2009) and preventing NLRC4 inflammasome activation (Higa *et al*., 2013).

**AopX** is related to the T3SS effector Plu4750 of *P. luminescens* which targets lipid raft in the cytoplasmic membrane (French *et al*., 2009) but its function is unknown.

We have also observed that **ExsE** and **Ati1** were secreted by the T3SS in wt SNs (P. Vanden Bergh, unpublished). In *P. aeruginosa*, it was shown that the T3SS secretion in extracellular medium and the T3SS translocation into host cell of ExsE was required for transcriptional induction of the T3SS (Urbanowski *et al*., 2007) (Fig. 1). It is not known whether ExsE plays a role within the host cell. **Ati1** is the only chaperone that is secreted by the T3SS (P. Vanden Bergh, unpublished) but it is not known whether it is also translocated into the fish cell.

**Figure 4:**
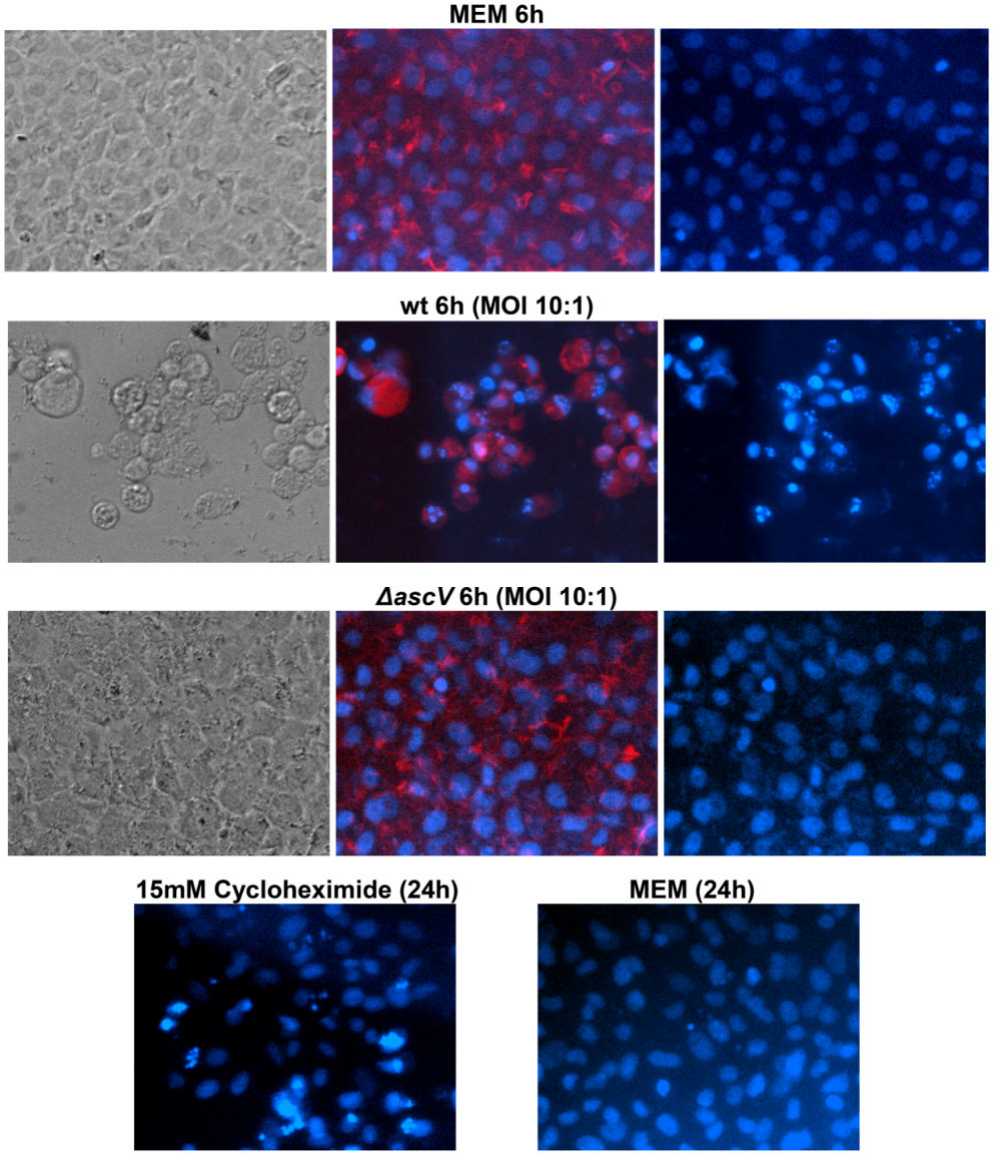
Morphological characteristics of the cytotoxicity induced by *A. salmonicida.* Besides the rapid cell-rounding due to the depolymerization of actin, *A. salmonicida* induces also rapid nuclear morphological changes characteristic of apoptosis (nuclear shrinkage and chromatin condensation). Epithelial fish cells (epithelioma papulosum cyprinid) were incubated 6 hours with wt (JF5054), *ΔascV* (JF2747) *A. salmonicida* (MOI of 10:1) or MEM medium (negative control). As a positive control of apoptosis, cells were incubated 24h with 15 mM cycloheximide. Blue colour represents nuclei stained with DAPI and red colour, actin stained with TRITC-phalloidin.

**Figure 5:**
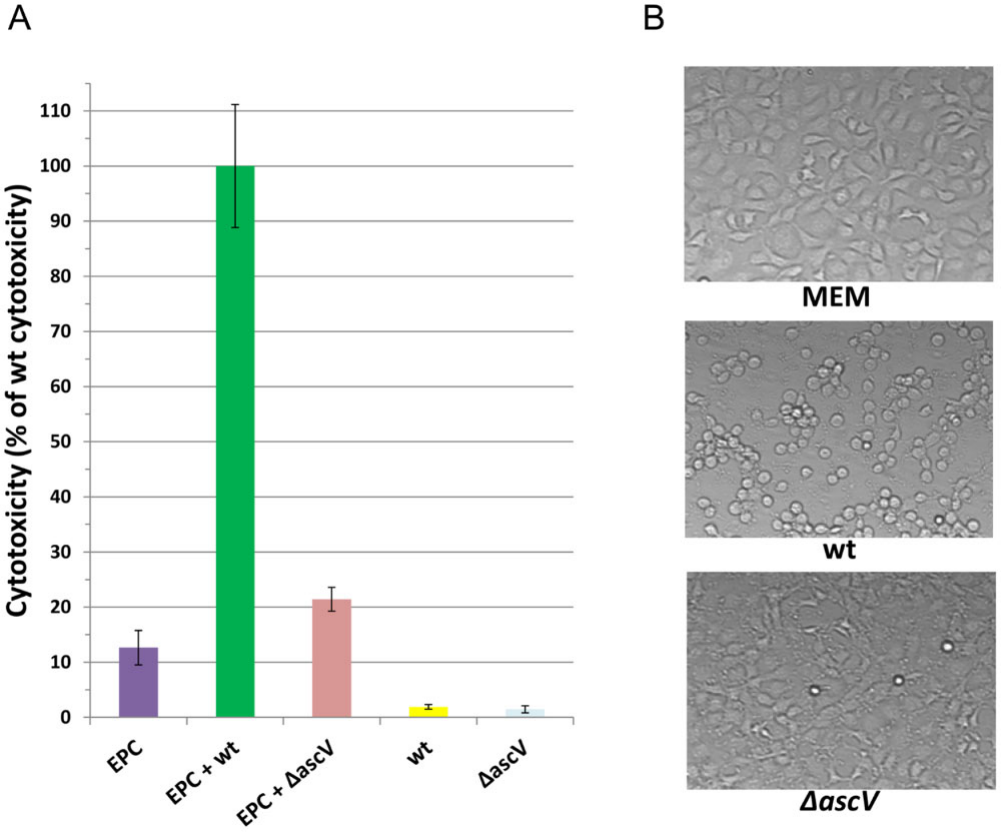
Membrane alterations induce by the *A. salmonicida* T3SS. A. Virulence of *A. salmonicida* wt (JF2267) and *ΔascV* (JF2747) mutant strains for EPC cells studied by LDH release assay after 3 hours of infection at an MOI of 100:1. Cell death is expressed as the percentage of maximal LDH release measured from lysed EPCs with the wt strain.B. Microscopic analysis of *A. salmonicida* wt and *ΔascV* mutant virulence for EPC cells one hour before the LDH release assay.

Interestingly, at least five effectors (AexT, Ati2, AopH, AopP and AopS) out of the eight that have been identified so far, exert different negative effects on cytoskeletal dynamics that could explain the rapid cell-rounding within less than 1 h observed when *A. salmonicida* is incubated with epithelial fish cells (Movies S1 and S2). The AopS function is redundant with the actin depolymerization effect of AexT and might be the reason why mutation seen in *aopS* has globally no consequence on the virulence of *A. salmonicida*. As an essential part of the cytoskeleton, actin is involved in a multitude of cellular processes that are keys to establish structure, morphology and motility of cells and cell components (Aktories *et al*., 2012): epithelium barrier function (establishment and maintenance of cell junctions and cell shape), motility and cytokinesis, muscle contraction, cell division, phagocytosis, signalling of immune cells, intracellular trafficking of vesicles and organelles. This prominent role in the eukaryotic homeostasis explains why actin proteins are prime targets for bacterial virulence factors (Aktories *et al*., 2012). In accordance with these observations, it is likely plausible that the actin depolymerization by the T3SS of *A. salmonicida* contributes to the colonization of the host and to the survival in phagocytic cells (Burr *et al*., 2005). Their ability to destabilize the actin cytoskeleton at different critical sites in the host cell could explain why the deletion of *aexT* alone (Fehr *et al*., 2007) or a triple knock-out mutant *ΔaexTΔaopOΔaopH* (Fast *et al*., 2009) are not sufficient to prevent the cell rounding and the virulent phenotype. Hence, a quadruple *ΔaexTΔaopOΔaopHΔati2* mutant would be expected to abolish the disruption of the actin cytoskeleton on the condition that the AopS gene is well inactivated. Besides the cell-rounding, another characteristic of cell death initiated by *A. salmonicida* is the rapid induction of nuclear morphological changes characteristic of apoptosis (nuclear shrinkage and chromatin condensation), which would be activated at least by the intracellular effect of AopP (Jones *et al*., 2012) (Fig. 4). At the end stage of the intoxication, the plasma membrane of completely disorganized host cells is alterated by the T3SS leading to the release of lactate dehydrogenase in the extracellular medium (P. Vanden Bergh, unpublished) (Fig. 5) as observed in *V. alginolyticus* (Zhao *et al*., 2011). This process could imply putative phospholipases (such as ASA_P5G088) thereby allowing the bacteria to damage the cell membrane and gain access to the nutrients that are released from the cytosol.

Besides these classical T3SS effectors, additional proteins with predicted T3SS secretion signal were excreted to a greater extent in wt SNs (EF-G, EF-Tu, DnaK, HtpG, PNPase, MdeA, PepN and OpdA) but to a clearly lesser extent than previously described T3SS effectors (P. Vanden Bergh, unpublished). These putative effectors show a high similarity with homologues present in eukaryotic cells, where they play fundamental roles and sometimes have alternative (moonlighting) functions: EF-1*α* for EF-Tu (Ejiri, 2002), HSP70 and HSP90 for DnaK and HtpG (Tsan and Gao, 2009; Singhto *et al*., 2013), eukaryotic aminopeptidases and thimet oligopeptidase for PepN and OpdA (Kessler *et al*., 2011). Some of them play a role in the virulence of other pathogens and are considered to be new targets for therapy (Neckers and Tatu, 2008; Barbier *et al*., 2013). It is tempting to assume that they too might be injected by *A. salmonicida* into host cells in order to interfere with these functions.

The proteomics study that we have conducted showed that weak amounts of T3SS effectors/translocators were found in *ΔascV* mutant SNs (AopH, AexT, AopD, Ati2, AopP, AopN, AopB and ExsE by order of decreasing importance), and the presence of these T3SS elements in mutant SNs was not due to bacterial lysis or cross-contaminations (P. Vanden Bergh, unpublished). The mutant strain thus continues to release weak amounts of Aops in SNs, either from the resting structural T3SS components or by an alternative secretion pathway. It is accepted that the T3SS arose from an exaptation of the flagellum (Abby and Rocha, 2012), and thus, it could be possible that FlhA (ASA_1351, polar flagella) and/or LfhA (ASA_0352, lateral flagella), showing 56% and 55% of similarity with AscV, respectively, might partially supply the function of this T3SS component. Another possibility is that a second mechanism of effectors/translocators secretion, clearly less efficient than the active T3SS, operates at the same time. Further investigations are therefore necessary to clarify the mechanism leading to the presence of effectors/translocators observed in the SN of the *ΔascV* mutant.

## The origin of the T3SS-induced virulence of *A. salmonicida* – A story of survival and adaptation

The adaptation of *A. salmonicida* T3SS effectors, shared by different environmental bacteria, to disrupt the host cytoskeleton is most intriguing, and it is tempting to hypothesize that these attack strategies are the result of a selective pressure to survive in the aquatic environment to the engulfment and killing by feral phagocytes, such as amoebas. The actin depolymerization could serve to paralyse the bacteriophagous protozoan, escape phagocytosis, survive inside the cell, spread in the environment and lyse these hosts to get an alternative source of aquatic nutriments. Hence, the well-known predator of bacteria would become a prey to be parasitized. Therefore, *A. salmonicida* strains virulent for fish have also been demonstrated to be virulent for amoeba (Paniagua *et al*., 2001; Daher *et al*., 2011), and it has been confirmed with our wt (JF2267) and *ΔascV* (JF2747) mutant strains that this cytotoxic effect was associated with the T3SS (Froquet *et al*., 2007). For example, in *V. parahaemolyticus*, it has been demonstrated that the T3SS-2 promotes survival of the bacterium in the interaction with diverse protist taxa and that the enhanced persistence is due to T3SS-2 effectors mediated cytotoxicity [some of which being homologous to *A. salmonicida* effectors (Table 1)] and facultative parasitism of *V. parahaemolyticus* on coexisting protists (Matz *et al*., 2011).

Because these wild phagocytes might serve as a reservoir and an amplifier for virulent *A. salmonicida*, it might explain why sediment is an important environmental reservoir of the bacterium as the pathogen can survive for a longer period of time and retain its pathogenicity in faecal and food waste sediment (Michel and Dubois-Darnaudpeys, 1980) at the bottom of sea cages, freshwater tanks or in pond mud (O’Brien *et al*., 1994). The high prevalence of protistan hosts in water with faecal contamination or plankton might serve the multiplication of virulent strains of *A. salmonicida* and could subsequently constitute sources of furunculosis outbreaks (King and Shotts, 1988; Nese and Enger, 1993).

## The entry of *A. salmonicida* into the fish host

**Figure 6:**
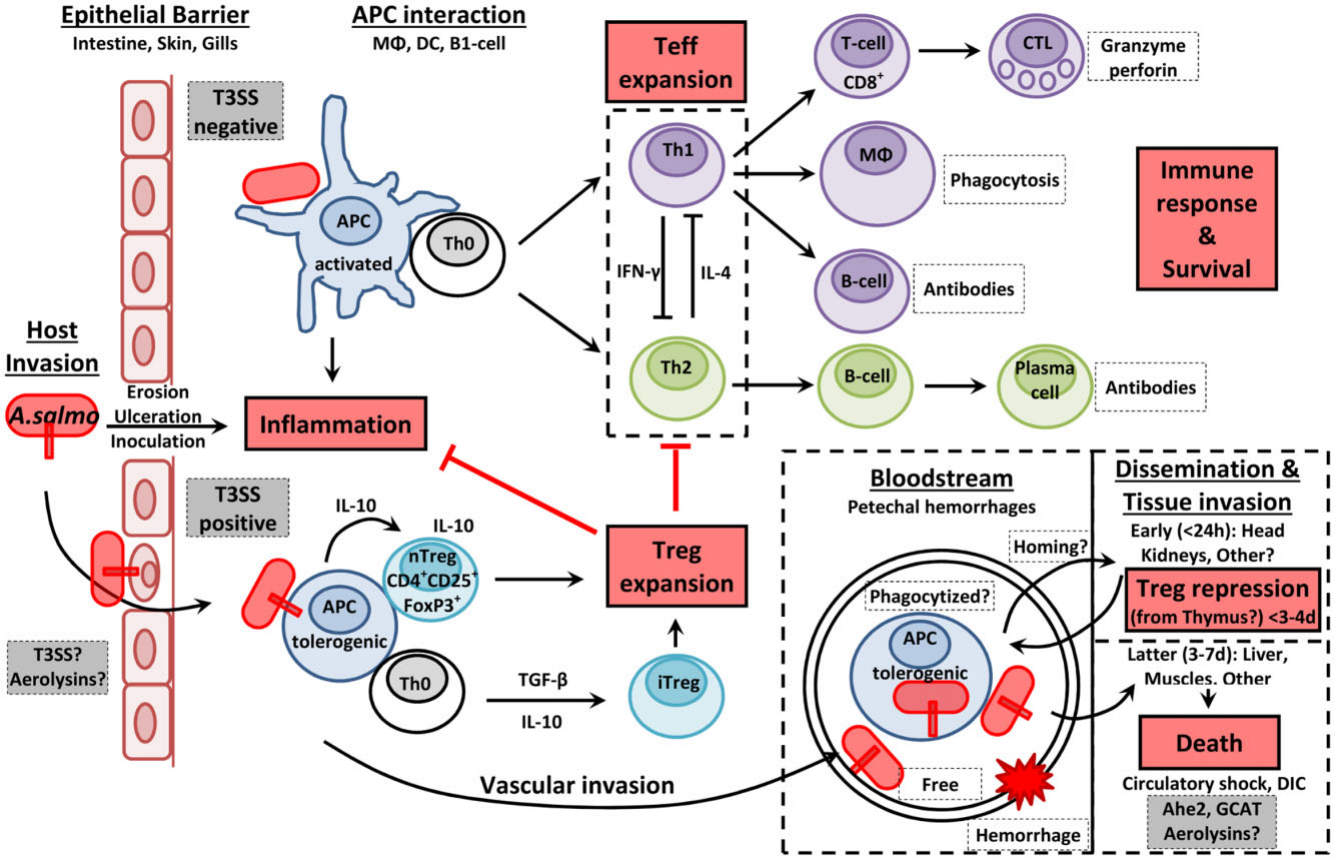
A model for *A. salmonicida* T3SS-dependent pathogenesis. We propose a model of pathogenesis in which T3SS^+^ *A. salmonicida* could hijack the induction of an efficient immune response by promoting tolerogenic DCs, IL-10 expression, Treg cells activation and expansion, and the inhibition of Teff (T and B cells) proliferation during the incubation period of the furunculosis. By promoting a pseudo tolerance state like a wolf in sheep’s clothing, some *A. salmonicida* bacteria could rapidly disseminate through the bloodstream (free or phagocytized) into major immune organs (e.g. the head kidneys) where they attack and neutralize APCs and B lymphocytes. Some Treg cells might directly come from the thymus in the head kidneys and repress the lymphoproliferation in a T3SS-dependent manner. Later in the disease, *A. salmonicida* would colonize the liver, the gills and lastly cardiac and skeletal muscles freed of leukocytes. After 3–5 days post-inoculation the capacity of T and B cells to proliferate (Dautremepuits *et al*., 2006) and the CTL activation [especially, in the head kidney (Kumari *et al*., 2013)] recover their functions but would appear too late to control efficiently the infection and protect fish from death. The cause of death would be associated with a septicemic state and circulatory shock. The inoculation of T3SS-negative *A. salmonicida* would induce a balanced Th1/Th2 protective immune response.

Several entry sites (skin, gills and the intestine) into fish have been described for *A. salmonicida* (Farto *et al*., 2011) but the route of infection would more likely be associated with any epithelial barrier injury (erosions/ulcerations) where the bacteria attach and penetrate into host tissues (Fig. 6). In order to initiate the disease, it could be a necessity for the bacterium to have direct access to epithelial cells and a hand-to-hand combat. Therefore, it could explain why challenges by immersion in laboratory facilities with healthy fish (intact epithelial barriers) and circulating fresh water are difficult even with high bacterial loads. This observation might support the idea that any stress perturbing mucus layer and/or epithelial integrity could be necessary to induce the furunculosis. Hence, it is frequent to read in the literature that in order to get mortality, the skin of fish is rubbed, scratched or wounded prior to an immersion challenge with *A. salmonicida*.

At the cell contact, the actin depolymerization induced by translocated T3SS effectors of *A. salmonicida* might already disturb the epithelium integrity (Movies S1 and S2) (Fig. 6). Aerolysins (AerA and AerB) might also participate in the epithelial barrier injury as described in *A. hydrophila* (Bucker *et al*., 2011). In experimental challenges of naïve trout with the wt virulent strain (JF5054), it was possible to get mortality by i.p. inoculations from 50 to 500 cfu per trout showing that the infectious dose can be very low if bacteria are in a state of high virulence. This fact raises the question of definition of a threshold to distinguish virulent and non-virulent *A. salmonicida* inocula. If the injection of hundreds of thousands of bacteria is necessary to induce a low level of mortality, does this mean that the *A. salmonicida* strain is really virulent and does it represent a natural infection?

## The T3SS and the escape from the immune response – The art of war

**Figure 7:**
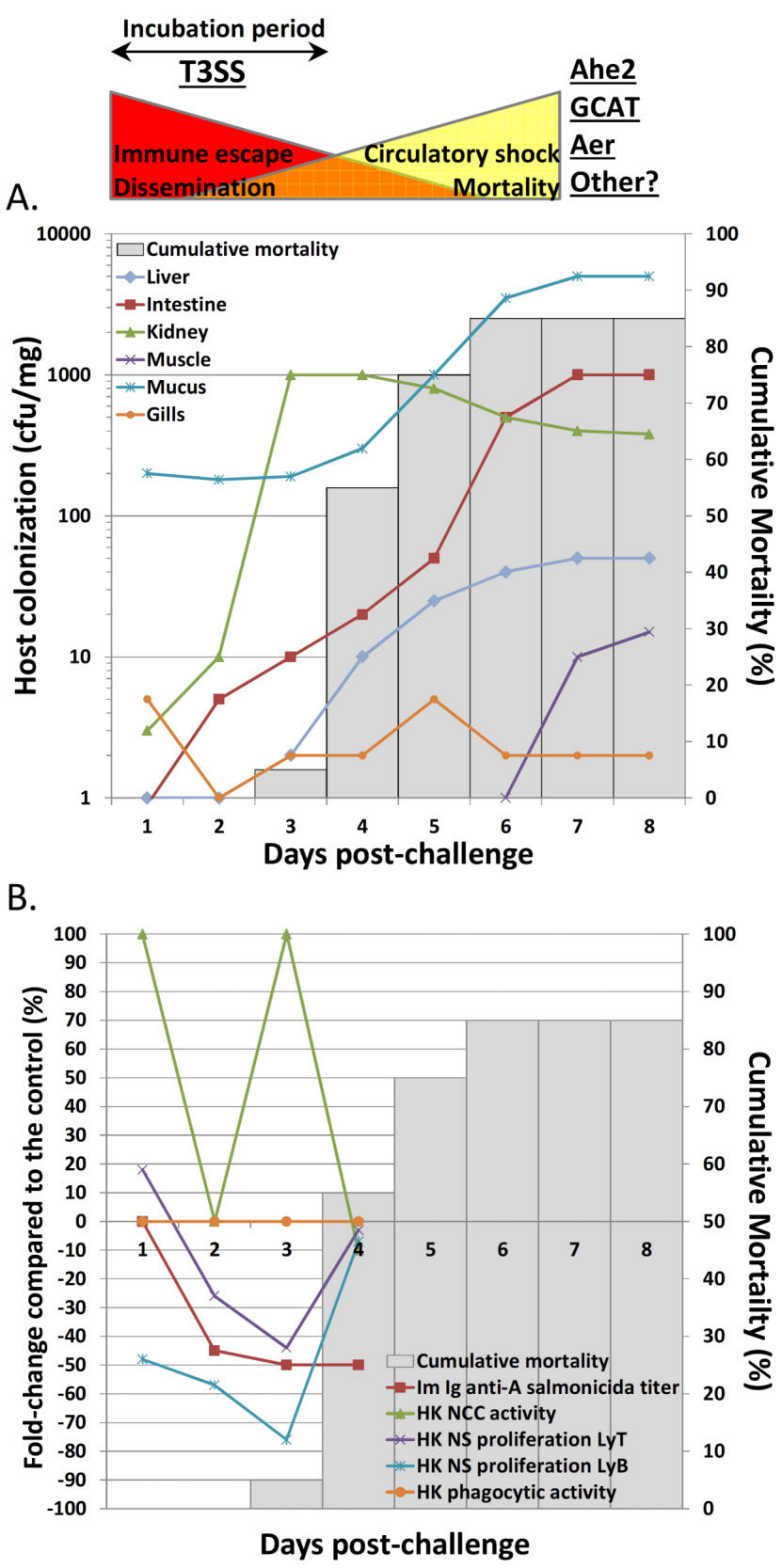
Evolution of host colonization, immune parameters and mortality during the incubation period of the furunculosis. In these figures, the authors used results from previous publications (Dautremepuits *et al*., 2006; Farto *et al*., 2011) to put the onset of fish mortality induced by *A. salmonicida* in perspective with tissue colonization (A) and immune parameters from the head kidney of fish (B). The incubation period of furunculosis could be dominated by the immunosuppressive effect of the T3SS leading to the dissemination of *A. salmonicida* in several organs while the later stages of the disease, associated to mortality and circulatory failure, would be dominated by the high production of other virulence factors such as Ahe2 serine protease and GCAT lipase. The hypothesized importance of *A. salmonicida* virulence factors is represented above figures. Diagram A represents tissue colonization of fish challenged with virulent T3SS^+^ *A. salmonicida* according to the results of Farto and collaborators (Farto *et al*., 2011). The kidney is the first organ massively invaded by *A. salmonicida*, as early as 12h post-challenge and without any symptoms, followed by the liver. Then, bacteria are isolated from muscles only in the later stages of the disease. Diagram B shows the behaviour of leukocytes from the head kidney of fish challenged with virulent T3SS^+^ *A. salmonicida* during the incubation period of furunculosis, according to the study of Dautremepuits and collaborators (Dautremepuits *et al*., 2006). The non-specific (NS) lymphoproliferation (B and T cells) is significantly inhibited in this organ during 3 days post-challenge and antibody titers specific of *A. salmonicida* are strongly depressed (Bricknell *et al*., 1999).

Once fish are challenged with *A. salmonicida*, a typical incubation period of 3–4 days occurs where bacteria rapidly disseminate (already 12 h post-challenge) in kidneys (Farto *et al*., 2011) (Fig. 7A). Then, *A. salmonicida* colonizes spleen, liver (Burr *et al*., 2005), and in the latest stage of the infection, the skeletal and cardiac muscles (Farto *et al*., 2011). In these sites, *A. salmonicida* gets access to its nutriments through cytolysis. The appearance of colonies in host tissues coincides with the mortality peak (Fig. 7A) (Burr *et al*., 2005; Farto *et al*., 2011). The incubation period is crucial for pathogenesis, and we hypothesize that virulent *A. salmonicida* strains use the T3SS and its effectors to neutralize the fish immune system along this period of the infection. In *Yersinia*, the inhibition of dendritic cell (DC) activation and T-cell proliferation by T3SS effectors are the mechanisms by which the bacterium evades innate and adaptative immune responses to generate the disease (Autenrieth *et al*., 2010). The scenario of a direct immunosuppressive effect induced by *A. salmonicida* was suspected by the Furunculosis Committee as early as in 1935 (Mackie *et al*., 1935), and thereafter, several observations have supported the hypothesis of an immunosuppressive mechanism similar to that of *Yersinia*:

1. the virulent T3SS^+^ JF2267 strain is able to survive phagocytosis by fish peripheral blood leukocytes *in vitro* (Burr *et al*., 2005),
2. the virulent T3SS^+^ 01-B526 strain depresses drastically B and T lymphoproliferation from head kidneys (HK) during the 3 days following the challenge and then return to the baseline at the mortality onset (Dautremepuits *et al*., 2006) (Fig. 7B),
3. there is no apparent leukocytic infiltration associated with colonies of T3SS^+^ *A. salmonicida* in fish tissues (absence of antigen-presenting cell activation and leukocyte migration) (Burr *et al*., 2005),
4. a drastic decrease in plasma antibody titres is immediately observed in the days following a challenge with virulent strains of *A. salmonicida* (Fig. 7B) (Bricknell *et al*., 1999; Romer *et al*., 2012). The ability of *A. salmonicida* to escape phagocytosis and inhibit lymphoproliferation might explain the hypoimmunoglobulinemia that could result from the combination of the inhibition of B lymphocyte differentiation/proliferation (blocking of antibody production) and the opsonization and formation of antigen/antibody complexes in the bloodstream (antibody depletion).

The mechanisms used by *A. salmonicida* to defeat the lymphocyte multiplication are currently not known but several observations tend to demonstrate that the bacterium may also induce tolerogenic DCs and activate suppressor regulatory T lymphocytes (Treg) in a T3SS-dependent manner (Fig. 6). In mammals, Treg cells in all lymphoid and intestinal organs are derived from thymus (Cebula *et al*., 2013). In many fish species, threads of cells directly connect the thymus with the HK, and this may be the route for lymphocyte migration from the thymus to the HK (Bowden *et al*., 2005). Interestingly, thymectomized fish injected with *A. salmonicida* show higher antibody titres than intact controls while the reconstitution of thymectomized fish with preserved thymocytes depresses the immunoglobulinemia at levels similar to those of the control group, and this suggests that *A. salmonicida* promotes an immunosuppressive cell population existing in this organ and acting on the B-cells (Findlay and Tatner, 1996). Moreover, IL-10 is a cytokine produced by various cell populations which downregulates the cellular immune response and contributes to Treg-mediated suppression in association with other cytokines (Yamaguchi *et al*., 2011). *In vitro*, virulent *A. salmonicida* elicits a significant increase in IL-10 expression by fish leukocytes from the HK whereas deletion of T3SS genes significantly decreases the expression of this cytokine (Fast *et al*., 2009). In mice infected with an AcrV-mutant of *A. hydrophila*, IL-10 levels were also significantly downregulated (Fadl *et al*., 2006). Furthermore, in the 3 days following a challenge with *A. salmonicida*, the expression of genes associated with the immunosuppressive Treg response such as Foxp3 (Zhang *et al*., 2011), fibroleukin Fgl2 (Millan *et al*., 2011; Liu *et al*., 2013), Es1 (HES1/KNPI) (Ostroukhova *et al*., 2006; Millan *et al*., 2011) and serum amyloid A (SAA) (Jensen *et al*., 1997; Nguyen *et al*., 2011) was enhanced in the HK of fish.

In non-infectious conditions, mammalian DCs that are present in gut, skin and lungs play a key regulatory mechanism of ‘tolerance’ to prevent excessive inflammation induced by the commensal flora. These tolerogenic DCs produce the immunomodulatory IL-10 cytokines and fail to deliver proper costimulatory signal to naïve CD4+ T-cell (Th0) for effector T (Teff) cells activation and proliferation. This results in T cell death, T cell anergy or induction and expansion of subsets of Treg cells (Kornete and Piccirillo, 2012). Specifically, Treg cells maintain order in the immune system by enforcing a dominant negative regulation on other immune cells. However, when a conventional bacterial pathogen (or T3SS negative *A. salmonicida*) penetrates host tissues (Fig. 6), immature DCs receive maturation signals through the pathogen-associated molecular patterns and damage associated molecular patterns receptors, and mature DCs initiate a three-step T cell activation process (Kornete and Piccirillo, 2012). In these conditions, naïve CD4+ T-cells (Th0) are then polarized towards Th1 and/or Th2 cells (depending on the stimulation and the cytokinic environment) to eliminate the pathogen.

In this view, it might be possible that *A. salmonicida* uses its T3SS to promote the tolerance pathways and hijack the immune response in the first days of the disease (Fig. 6). For example, LcrV (homologous to AcrV) in *Yersinia* induces immunosuppression by promoting the differentiation of tolerogenic DCs via the interaction with TLR2/TLR6 and CD14 receptors (DePaolo *et al*., 2008) and the amplified release of IL-10 from host cells (Reithmeier-Rost *et al*., 2004). The exacerbated release of IL-10 by *Yersinia* plays a crucial role in the pathogenesis since IL-10-deficient mice are resistant to the infection (Sing *et al*., 2002a). At least two regions of LcrV were associated with the induction of the IL-10 immunomodulatory responses (Sing *et al*., 2002b; Overheim *et al*., 2005). In *A. salmonicida*, AcrV shows a high conservation (66% and 93% of similarity) with these fragments. Hence, these conserved regions could play the same IL-10 dependent immunomodulatory function in AcrV.

Moreover, although virulent *A. salmonicida* are detected in the HK as early as 12 h post-challenge by immersion (Farto *et al*., 2011), the recovery of the lymphoproliferation (Dautremepuits *et al*., 2006) and the expression of genes associated with a Th1 response and the activation of cytotoxic T lymphocytes (CTL) (such as IFN-γ and granzyme) occur only 3–4 days after the inoculation (Millan *et al*., 2011; Kumari *et al*., 2013) (Fig. 7B) and do not appear to be sufficient to save fish from death. During this incubation period, *A. salmonicida* might thus trigger the migration of activated Treg cells from the thymus directly into the infected HK inhibiting the lymphocyte proliferation in this organ (Bowden *et al*., 2005).

The induction of the CTL response in HKs could be associated with the multiplication of *A. salmonicida* in this organ, the repression of the T3SS and the induction of proteases, lipases expression by the QS. It can also be associated with the observation that *A. salmonicida* is able to invade and survive within HK leukocytes (Fast *et al*., 2009), and would therefore induce an immune response specific to intracellular pathogens. The HK hosts diverse antigen-presenting cell (APC) types (melanomacrophages, B cells, reticular cells) and is thus suspected to serve as a major secondary lymphoid organ to which APCs loaded with antigens in the peripheral organs migrate (homing) (Iliev *et al*., 2013). This might suggest that *A. salmonicida* uses APCs and the homing to rapidly disseminate in the lymphoid system which explains that these organs are the first to be infected (Farto *et al*., 2011) (Fig. 7A). In this view, *A. salmonicida* has been demonstrated to be able to survive and even replicate inside fish non-phagocytic cells and macrophages, thereby supporting the notion that the bacterium can be a facultative intracellular pathogen (Garduno *et al*., 1993; 2000). This process would be primordial for *A. salmonicida* to survive *in vivo* soluble lytic activity present in extracellular fluids (Garduno *et al*., 2000).

The rapid attack and neutralization of immune functions in the early stages of the disease may explain the absence of leukocyte migration in other infected tissues in the final stage of the furunculosis. Peracute infections most often occur in fingerling fish which die from shock without showing marked clinical manifestations. Acute infections generally occur in juvenile and adult fish and are associated with haemorrhages in all organs. *Aeromonas salmonicida* ultimately induces a septicemic state but teleost fish are resistant to endotoxic shock (Sepulcre *et al*., 2009), and the cause of death in furunculosis is more likely due to a shock induced by vascular failure associated with disseminated intravascular coagulation, a consumptive coagulopathy that may rapidly be induced at least by the intravascular injection of the serine protease Ahe2 (AspA) (Salte *et al*., 1993) and the LPS-activated GCAT lipase (SatA) (Salte *et al*., 1992). Thus, the early stage of the furunculosis could be dominated by the T3SS inducing immune escape and dissemination of a few bacteria in lymphoid organs. Thereafter, the later stages of the disease would be associated with a large multiplication and bacterial dissemination in all organs, ultimately resulting in circulatory shock due to other virulence factors (Fig. 7).

## The protective immune response to *A. salmonicida*

Various studies tend to demonstrate that the protective immune response against *A. salmonicida* would result from a balanced Th1/Th2 response (Fig. 6). For example, fish protection is correlated with a high level of circulating antibodies specific to *A. salmonicida* (Romer *et al*., 2012; Romstad *et al*., 2013). The administration to fish of specific immunostimulants such as CpG oligodeoxynucleotides or the heptanoyl tripeptide FK-565 which indirectly enhance the activation of Th1 cells (Krieg, 2002; Tada *et al*., 2005) increases their survival following a challenge with a virulent strain of *A. salmonicida* (Kitao and Yoshida, 1986; Carrington and Secombes, 2007) and suggest that promoting a Th1 polarization might be important to overcome the infection.

Like other pathogens using the T3SS for their virulence, we suspect that *A. salmonicida* might use certain strategies to interfere with the endogenous pathway (MHC-I) and the ultimate activation of CD8^+^ T-cells to avoid the destruction of its Trojan horse (APC) by CTLs. Actually, it is accepted that T3SS effectors translocated into the host cytoplasm should be, like viral proteins, perfect candidates for antigen presentation by the endogenous pathway which canonically eliminate cells containing the intracellular pathogen (Mantegazza *et al*., 2013). Bergman and colleagues (2009) showed that the CTL response played a critical role in eliminating *Yersinia* infections, and that this response was directed against Yop T3SS effectors. In *Salmonella*, MHC class-I-restricted CD8 T cells can play a protective role during primary *Salmonella* infection (Lee *et al*., 2012). Moreover, *Salmonella* T3SS effectors have been successfully used for vaccination strategy specifically through the MHC-I/CD8^+^ T-cell pathway (Hegazy *et al*., 2012). Eliciting an immune response against *A. salmonicida* through the endogenous pathway of antigen presentation might thus be an interesting strategy for vaccine approaches. However, it was also predicted by bioinformatics that T3SS effectors with homologues in *A. salmonicida* show clear escape mutations and have low epitope densities for MHC, suggesting that these bacteria, like viruses, are evolutionarily selected to ensure their survival in the presence of CD8^+^ T-cells (Maman *et al*., 2011). For example, while YopE, a homologue to AexT, was not protective in experiments using the whole protein as antigen, the N-terminal domain (YopE69-77) revealed to be a major protective antigen eliciting CD8 T-cell immunity (Zhang *et al*., 2012). In spite of the fact that most of the T3SS effectors are immune suppressor/modulator, this result shows that they can contain protective epitopes that might be promising candidates for vaccination. In this view, it has been demonstrated that polymorphisms in MHC-II, but also MHC-I, were significantly associated with resistance of Atlantic salmon to furunculosis (Grimholt *et al*., 2003; Kjoglum *et al*., 2008) and highlight the importance of the endogenous pathway of antigen presentation in this disease.

Vaccine and therapeutic approaches stopping the T3SS-dependent immunosuppressive strategies of *A. salmonicida* at the onset of the disease might thus represent the best protection of fish against furunculosis. According to this hypothesis, we recently observed that immunization of fish with bacterins of the *ΔascV* mutant strain of *A. salmonicida* (JF2747) that expresses low levels of all the T3SS components (immunosuppressors), induced a better protection (25% increase in the survival) than vaccination with the wt strain JF5054 expressing the T3SS at high levels (Vanden Bergh *et al*., 2013). This result was unexpected but it supports our current model of pathogenesis, and challenged the hypothesis that mounting specific antibodies against proteins of the T3SS yields better protection. At the onset of a furunculosis outbreak, therapies targeting either the inhibition of the T3SS expression (such as pro-quorum-sensing molecules) (Ng *et al*., 2012) or secretion (Duncan *et al*., 2012) by *A. salmonicida*, or promoting the Th1 immune response (Krieg, 2002; Tada *et al*., 2005), or specifically inhibiting the Treg expansion (Casares *et al*., 2010) are expected to help resolving the disease and constitute alternatives to classical antibiotics.

## Conclusions

The typical furunculosis induced by *Aeromonas salmonicida* subsp. *salmonicida* is a disease that has been known for over a century. The mechanisms of virulence were poorly understood until the discovery of the T3SS as a major virulence system, a decade ago. In this review, we have proposed a comprehensive model of pathogenesis for *A. salmonicida* involving the various actions of T3SS and its effectors on the morphogenesis, the vital functions and the immune defence of the host cells. This concerted action of virulence attributes permits the pathogen to cause disease in salmonid fish by circumventing all barriers imposed by the host and the environment. Although further investigations both on the pathogen and on the host will be necessary to fully confirm its validity, this knowledge provides strategies in designing novel preventive and therapeutic approaches to optimize environmentally and economically sustainable fish farming.

## Supporting Information

Figure S1 Genetic comparison of T3SSs from the Ysc family. Comparison of the organization of T3SS clusters in *A. salmonicida* subsp. *salmonicida* A449, *A. hydrophila* SSU, *A. veronii* AER39, *A. diversa* 2478-85, *Pseudomonas aeruginosa* PAO1, *Photobacterium damselae* subsp. *damselae* CIP 102761, *Photorhabdus luminescens* subsp. *laumondii* TTO1, *Yersinia pestis* biovar Antiqua str. E1979001, and *Vibrio parahaemolyticus* RIMD 2210633.

Table S1 Profound analysis and comparison of published Aeromonas genomes. Grey: conserved ORFs; light green: ORFs specific of the species; yellow: IS630; pink: other IS elements; red: putative or characterized virulence factors; mauve: ORFs for resistance to antibiotic or heavy metal; dark green: ORFs associated to pili, fimbriae or flagella; blue: ORFs associated with phage; cyan: tRNA and rRNA; orange: ORFs with homology to eukaryotic genes. *Aeromonas salmonicida* subsp. *salmonicida* A449, *A. salmonicida* subsp. achromogenes AS03, *A. salmonicida* (ATCC7966, ML09-119 and SSU), *A. caviae* Ae398, *A. veronii* (B565, AMC34, AMC35, AER39 and AER397), *A. aquariorum* AAK1, and *A. diversa* 2478-85.

Movies S1 and S2 Rapid cell-rounding induced by the T3SS of *A. salmonicida*. Epithelioma papulosum cyprini cells (EPCs) seeded in a glass bottom well and exposed to *A. salmonicida* wt JF2267 (S1) and ΔascV mutant JF2747 (S2) expressing GFP (after induction with IPTG) for 120 min. Following infection at 20°C, time-lapse imaging using video microscopy was performed using a Nikon Eclipse TE2000-U inverted microscope equipped with a climate-controlled chamber. Data acquisition and image processing were performed using NIS software of Nikon Instruments. DIC and fluorescence images were acquired and assembled in movies. Image acquisition at intervals of 2 min for 120 min. MOI of 10:1 (bacteria: fish cells).
