# Supplementary material for: Aeromonas salmonicida subsp. salmonicida in the light of its type-three secretion system
Source: Microb Biotechnol. 2013 Oct 7;7(5):381–400. doi: 10.1111/1751-7915.12091 (PMC4229320; doi:10.1111/1751-7915.12091)
Supplement: Figure S1 — Genetic comparison of T3SSs from the Ysc family. Comparison of the organization of T3SS clusters in A. salmonicida subsp. salmonicida A449, A. hydrophila SSU, A. veronii AER39, A. diversa 2478-85, Pseudomonas aeruginosa PAO1, Photobacterium damselae subsp. damselae CIP 102761, Photorhabdus luminescens subsp. laumondii TTO1, Yersinia pestis biovar Antiqua str. E1979001, and Vibrio parahaemolyticus RIMD 2210633. [file mbt20007-0381-sd1.pptx]

## Slide 1
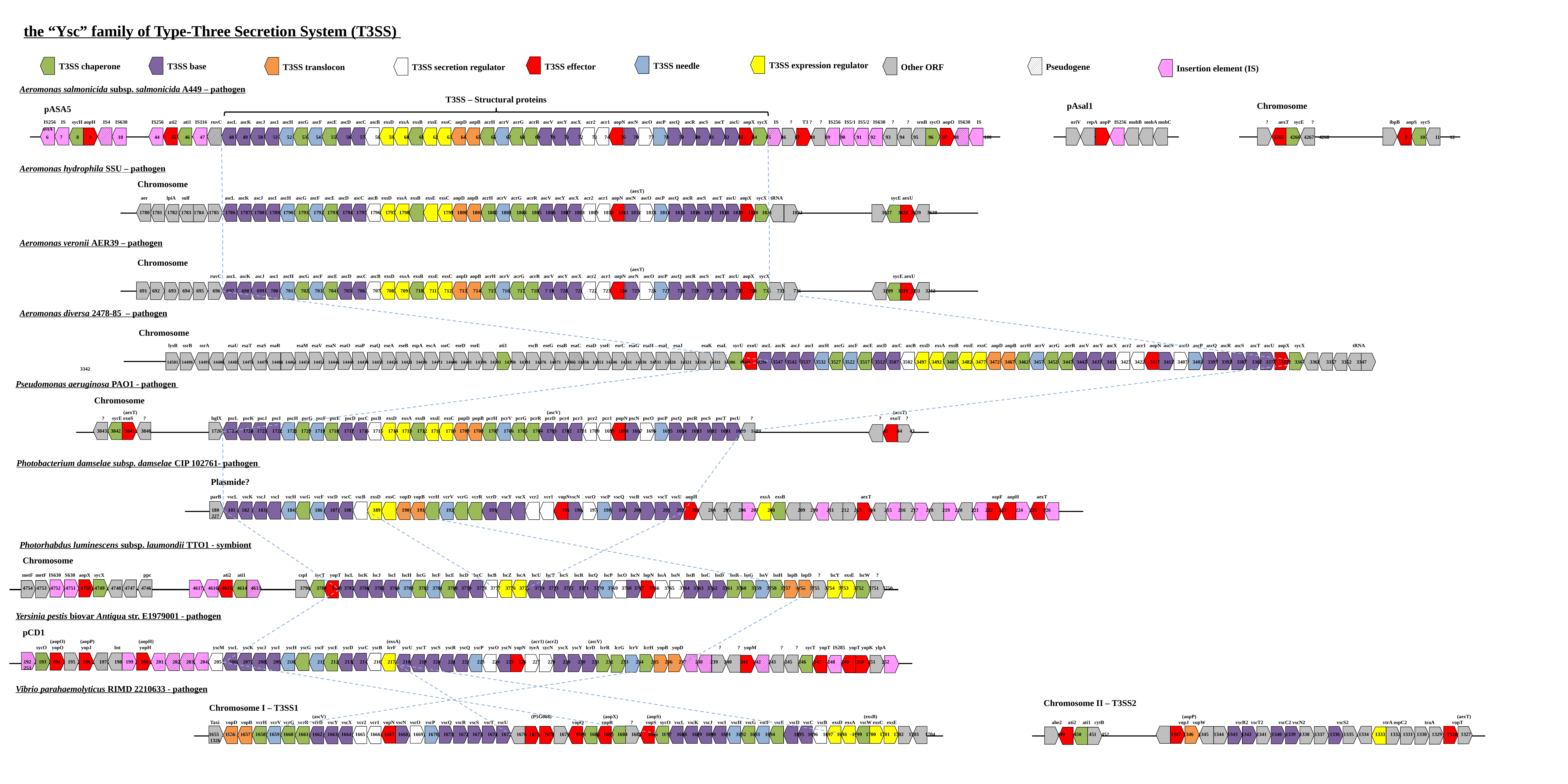

the “Ysc” family of Type-Three Secretion System (T3SS)
T3SS expression regulator
T3SS needle
T3SS chaperone
T3SS base
T3SS effector
Pseudogene
T3SS translocon
Other ORF
T3SS secretion regulator
Insertion element (IS)
Aeromonas salmonicida subsp. salmonicida A449 – pathogen
T3SS – Structural proteins
pAsal1
Chromosome
pASA5
 IS256 IS sycH aopH IS4 IS630 IS256 ati2 ati1 IS116 ruvC ascL ascK ascJ ascI ascH ascG ascF ascE ascD ascC ascB exsD exsA exsB exsE exsC aopD aopB acrH acrV acrG acrR ascV ascY ascX acr2 acr1 aopN ascN ascO ascP ascQ ascR ascS ascT ascU aopX sycX IS ? T3 ? ? IS256 IS5/1 IS5/2 IS630 ? ? srnB sycO aopO IS630 IS oriV repA aopP IS256 mobB mobA mobC ? aexT sycE ? ibpB aopS sycS avtA
 6 7 8 9 10 44 45 46 47 48 49 50 51 52 53 54 55 56 57 58 59 60 61 62 63 64 65 66 67 68 69 70 71 72 73 74 75 76 77 78 79 80 81 82 83 84 85 86 87 88 89 90 91 92 93 94 95 96 97 98 100 4265 4266 4267 4268 9 10 11 12
Aeromonas hydrophila SSU – pathogen
Chromosome
 				 (aexT)
 aer lpiA sulf ascL ascK ascJ ascI ascH ascG ascF ascE ascD ascC ascB exsD exsA exsB exsE exsC aopD aopB acrH acrV acrG acrR ascV ascY ascX acr2 acr1 aopN ascN ascO ascP ascQ ascR ascS ascT ascU aopX sycX tRNA sycE aexU
1780 1781 1782 1783 1784 1785 1786 1787 1788 1789 1790 1791 1792 1793 1794 1795 1796 1797 1798 1799 1800 1801 1802 1803 1804 1805 1806 1807 1808 1809 1810 1811 1812 1813 1814 1815 1816 1817 1818 1819 1820 1821 1822 3627 3628 3629 3630
Aeromonas veronii AER39 – pathogen
Chromosome
 				 (aexT)
 ruvC ascL ascK ascJ ascI ascH ascG ascF ascE ascD ascC ascB exsD exsA exsB exsE exsC aopD aopB acrH acrV acrG acrR ascV ascY ascX acr2 acr1 aopN ascN ascO ascP ascQ ascR ascS ascT ascU aopX sycX sycE aexU
691 692 693 694 695 696 697 698 699 700 701 702 703 704 705 706 707 708 709 710 711 712 713 714 715 716 717 718 7 19 720 721 722 723 724 725 726 727 728 729 730 731 732 733 734 735 736 3209 3210 3211 3212
Aeromonas diversa 2478-85 – pathogen
Chromosome
 lysR ssrB ssrA esaU esaT esaS esaR esaM esaV esaN esaO esaP esaQ eseA eseB espA escA sseC eseD eseE ati1 escB eseG esaB esaC esaD yseE esrC esaG esaH esaI esaJ esaK esaL sycU exoU ascL ascK ascJ ascI ascH ascG ascF ascE ascD ascC ascB exsD exsA exsB exsE exsC aopD aopB acrH acrV acrG acrR ascV ascY ascX acr2 acr1 aopN ascN ascO ascP ascQ ascR ascS ascT ascU aopX sycX tRNA
 14501 14496 14491 14486 14481 14476 14471 14466 14461 14456 14451 14446 14441 14436 14431 14426 14421 14416 14411 14406 14401 14396 14391 14386 14381 14376 14371 14366 14356 14351 14346 14341 14336 14331 14326 14321 14316 14311 14306 14301 14296 3547 3542 3537 3532 3527 3522 3517 3512 3507 3502 3497 3492 3487 3482 3477 3472 3467 3462 3457 3452 3447 3442 3437 3432 3427 3422 3417 3412 3407 3402 3397 3392 3387 3382 3377 3372 3367 3362 3357 3352 3347 3342
Pseudomonas aeruginosa PAO1 - pathogen
Chromosome
 (aexT) (ascV) (aexT)
 ? sycE exoS ? bglX pscL pscK pscJ pscI pscH pscG pscF pscE pscD pscC pscB exsD exsA exsB exsE exsC popD popB pcrH pcrV pcrG pcrR pcrD pcr4 pcr3 pcr2 pcr1 popN pscN pscO pscP pscQ pscR pscS pscT pscU ? ? exoT ?
 3843 3842 3841 3840 1726 1725 1724 1723 1722 1721 1720 1719 1718 1717 1716 1715 1714 1713 1712 1711 1710 1709 1708 1707 1706 1705 1704 1703 1702 1701 1700 1699 1698 1697 1696 1695 1694 1693 1692 1691 1690 1689 45 44 43
Photobacterium damselae subsp. damselae CIP 102761- pathogen
Plasmide?
parB vscL vscK vscJ vscI vscH vscG vscF vscD vscC vscB exsD exsC vopD vopB vcrH vcrV vcrG vcrR vcrD vscY vscX vcr2 vcr1 vopNvscN vscO vscP vscQ vscR vscS vscT vscU aopH exsA exsB aexT ospF aopH aexT
180 181 182 183 184 186 187 188 189 190 191 192 193 195 196 197 198 199 200 201 202 203 204 205 206 207 208 209 210 211 212 213 214 215 216 217 218 219 220 221 222 223 224 225 226 227
Photorhabdus luminescens subsp. laumondii TTO1 - symbiont
Chromosome
 metF metF IS630 S630 aopX sycX ppc ati2 ati1 cspI sycT yopT lscL lscK lscJ lscI lscH lscG lscF lscE lscD lscC lscB lscZ lscA lscU lscT lscS lscR lscQ lscP lscO lscN lopN lssA lssN lssB lssC lssD lssR lssG lssV lssH lopB lopD ? lscY exsE lscW ?
4754 4753 4752 4751 4750 4749 4748 4747 4746 4617 4616 4615 4614 4613 3790 3789 3788 3787 3786 3785 3784 3783 3782 3781 3780 3779 3778 3777 3776 3775 3774 3773 3772 3771 3770 3769 3768 3767 3766 3765 3764 3763 3762 3761 3760 3759 3758 3757 3756 3755 3754 3753 3752 3751 3750
Yersinia pestis biovar Antiqua str. E1979001 - pathogen
pCD1
 (aopO) (aopP) (aopH) (exsA) (acr1) (acr2) (ascV)
 sycO yopO yopJ Int yopH yscM yscL yscK yscJ yscI yscH yscG yscF yscE yscD yscC yscB lcrF yscU yscT yscS yscR yscQ yscP yscO yscN yopN tyeA sycN yscX yscY lcrD lcrR lcrG lcrV lcrH yopB yopD ? ? yopM ? ? sycT yopT IS285 yopT yopK ylpA
192 193 194 195 196 197 198 199 200 201 202 203 204 205 206 207 208 209 210 211 212 213 214 216 217 218 219 220 221 222 223 224 225 226 227 228 229 230 231 232 233 234 235 236 237 238 239 240 241 242 243 245 246 247 248 249 250 251 252 253
Vibrio parahaemolyticus RIMD 2210633 - pathogen
Chromosome II – T3SS2
Chromosome I – T3SS1
 (ascV) (P5G088) (aopX) (aopS) (exsB) (aopP) (aexT)
 Taxi vopD vopB vcrH vcrV vcrG vcrR vcrD vscY vscX vcr2 vcr1 vopN vscN vscO vscP vscQ vscR vscS vscT vscU vopQ vopR ? vopS sycO vscL vscK vscJ vscI vscH vscG vscF vscE vscD vscC vscB exsD exsA vscW exsC exsE ahe2 ati2 ati1 cytB vopJ vopW vscR2 vscT2 vscC2 vscN2 vscS2 vtrA ospC2 traA vopT
1655 1656 1657 1658 1659 1660 1661 1662 1663 1664 1665 1666 1667 1668 1669 1670 1671 1672 1673 1674 1675 1676 1677 1678 1679 1680 1682 1683 1684 1685 1686 1687 1688 1689 1690 1691 1692 1693 1694 1695 1696 1697 1698 1699 1700 1701 1702 1703 1704 449 450 451 452 1347 1346 1345 1344 1343 1342 1341 1340 1339 1338 1337 1336 1335 1334 1333 1332 1331 1330 1329 1328 1327 1326
